# To ChatGPT or not to ChatGPT: the use of artificial intelligence in writing scientific papers

**DOI:** 10.1093/braincomms/fcad266

**Published:** 2023-11-01

**Authors:** Manuela Marescotti

**Affiliations:** Edinburgh, UK

## Abstract

Our Scientific Editor discusses the current use of artificial intelligence in writing academic papers and reports the updated guidelines for *Brain Communications* on the use of this tool in scientific writing.

## Graphical Abstract

**Graphical Abstract:**
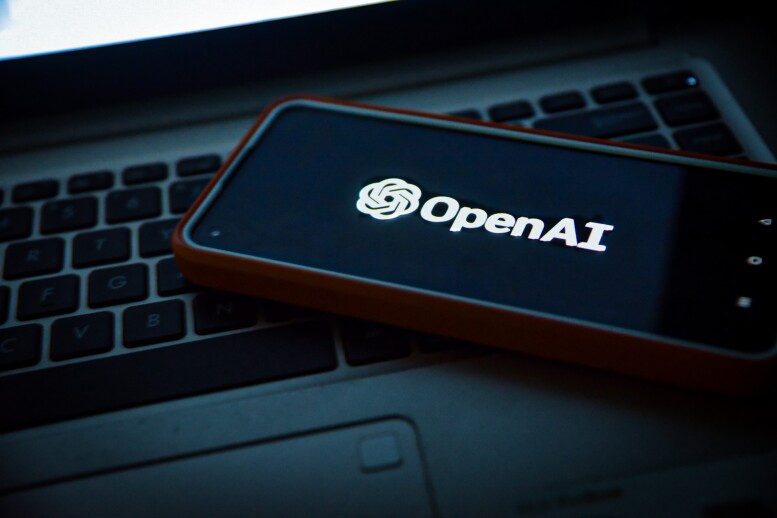

During the past 10 months, the global landscape has been profoundly influenced by the rise of artificial intelligence (AI) for generating textual content, graphics, and various digital materials. Within this transformative context, the scientific community, in particular, has felt a significant impact, as AI appeared to become a valuable ally in the creation of academic documents.

In November 2022, the well-known AI chatbot, ChatGPT (Generative Pretrained Transformer), became accessible to the public for free.^1^ This technology is capable of generating coherent text in various formats, including poetry, prose, computer code, and edited versions of input text.^1^ Therefore, this tool emerged to be potentially useful for grant writing and academic article composition.

Researchers have tested ChatGPT’s capabilities in the context of enhancing papers,^2^ hoping to speed up a process that is usually very time-consuming for both native and non-native English speakers. A consensus is forming regarding the limitations of this tool in terms of generating knowledge, stemming from two primary factors: the tool’s construction methodology and its lack of transparency regarding information sources.^1^ ChatGPT generates responses based on statistical language patterns extracted from online databases, without discerning the accuracy of the information. In addition, the absence of references for the generated content poses challenges when assessing its reliability.^3^ For these reasons, it is imperative that individuals using this tool are able to verify the accuracy of its responses.

It is worth noting that in recent months, preprint server administrators reported some submissions where the AI chatbot ChatGPT has been included as a co-author.^4^ This practice has raised concerns within the scientific community, particularly among academic journal editors and publishers. Most scientific publishers adhere to the Committee on Publication Ethics guidelines around authorship, which state that all listed authors must make a substantial contribution to the work and be accountable for the work that was done and its presentation in a publication.^5^ The use of AI does not meet these criteria since a chatbot cannot take responsibility as it is not an individual. Thus, including ChatGPT in the author list of a paper may go against the fundamental rules of authorship in academic writing, and fall into misconduct. To avoid that, it is paramount to establish clear guidelines regarding the appropriate acknowledgement of the tool’s use in academic writing.

Throughout 2023, academic journal editors, publishers, and preprint server administrators have progressively recognized the need to revise their author guidelines concerning the use of ChatGPT and similar AI tools in article composition, all with the goal of maintaining the quality standards of their published materials. Notably, *Brain Communications* has also revised its guidelines on this issue (https://academic.oup.com/braincomms/pages/General_Instructions), taking a stance on the reporting of AI usage in manuscript creation. Our stance follows the World Association of Medical Editors’ recommendations on chatbots and scholarly manuscripts. If an AI such as a chatbot or similar program is used in the development of a paper, we require:

- A Large Language Model (LLM), chatbot, or other AI cannot be credited as an author, as authorship requires that the author be accountable for the submitted/published work, and AI cannot fulfil this requirement of authorship.
- Authors listed on the paper must review the content generated by the LLM and take full responsibility for it, as they would for any other content within the submitted/published work.
- The use of LLM tools must be documented in the Methods, Acknowledgements, or another appropriate section of the paper.

Aside from the ethical considerations outlined above, the use of chatbots for generating text largely generates poor-quality outputs. As an experiment, I asked chatGPT to ‘Write an Editorial about the involvement of ChatGPT in writing a peer-reviewed article. Also, discuss the position of the main neuroscientific peer-reviewed journals about ChatGPT. This editorial has to be a maximum of 1500 words long and it should have no more than 10 references’. Our editorial team all agreed that while the output raised several good points, it was not scholarly, as it was quite repetitive and did not cite any verifiable sources of information. We were quite amused at how positive ChatGPT is about itself. Here is a snippet of the AI-generated text:

In recent years, artificial intelligence has made remarkable strides in various fields, and the realm of scientific research is no exception. One of the most fascinating developments in this regard has been the integration of AI-powered language models, such as ChatGPT, into the process of generating scientific content. The collaboration between human researchers and AI models like ChatGPT has led to the creation of peer-reviewed articles in various disciplines, including neuroscience. […..] ChatGPT, developed by OpenAI, represents a cutting-edge example of AI language models. It has been trained on a vast corpus of text, allowing it to generate coherent and contextually relevant responses to a wide range of prompts. While initially designed for chatbot applications, it has found utility in assisting humans in various tasks, including scientific writing.

In summary, while we recognize the utility of chatbots for editing grammar and syntax, we do not recommend using them to generate text for your papers!

The cover image for this issue illustrates three main elements of a study by Huang *et al*.^6^ on genetic risk factors for dementia: cortical morphology from high-resolution 3T MRI, genetic risk factors of late-onset dementia, and use of machine learning.
